# Choking Hazards: Are Current Product Testing Methods for Small Parts Adequate?

**DOI:** 10.1155/2017/4705618

**Published:** 2017-05-28

**Authors:** Athena Neofotistos, Nancy Cowles, Ragini Sharma

**Affiliations:** ^1^Roosevelt University, Chicago, IL, USA; ^2^Kids In Danger, Chicago, IL, USA

## Abstract

Choking on small parts remains one of the leading causes of death and injury in infants and toddlers. The current method of testing for small parts, created by the Consumer Product Safety Commission (CPSC), has become outdated and has yet to be changed despite the many deaths and injuries of children. The method uses a device called the small parts test fixture (SPTF) that is supposed to mimic the size of a fully expanded throat of a toddler. If a product does not fit inside the cavity of the SPTF, then it is deemed safe to play with because it “will not fit” in the esophagus of a child. The present study obtains a dataset of products recalled by the CPSC within the last twelve years due to choking hazards/incidents and discovers that a noteworthy amount of the children's products have parts that are larger than the fixture size and are still capable of causing choking. This study indicates that a larger SPTF size must be implemented by the CPSC in order to prevent future choking incidents on small parts.

## 1. Introduction

Choking on small parts is one of the leading, yet preventable causes of injury and death in infants and small children [1]. Children's products contain small parts or release small parts through detachment or breakage during normal play, which pose choking hazards and may be undetectable to parents and caregivers. Small parts have caused choking-related deaths in over 90 children between 2001 and 2012, as cited in the 28th Annual Survey of Toy Safety [2]. Limiting the prevalence of choking and related occurrences is an important public health goal. The current federal rule requiring the testing of small parts protects children under three years of age, which is the age group most likely to mouth objects [3]. The risk of choking can potentially be decreased by requiring that products for children under the age of three meet a new standard for size and breakability.

The small parts test regulation is required by the Federal Hazardous Substances Act under the Code of Federal Regulation (CFR), premised to prevent death and injuries to children under the age of three from choking on, inhaling, or swallowing small objects they may put in their mouths [4]. The current testing method to prevent choking hazards employs an apparatus dubbed the small parts test fixture (SPTF), created by the US Consumer Product Safety Commission (CPSC). The SPTF measures whether a toy is too large to enter a child's esophagus and, thus, can be played with safely.

If the object fits inside the cavity of the SPTF, it is too small and can potentially be lodged inside the throat of the child and cause choking. The SPTF measures 1 inch to 2.25 inches in height, slanted on a diagonal plane, and 1.25 inches in diameter at its current size. According to the American Academy of Pediatrics, this range would make choking on a small part highly improbable as it approximates the size of a fully expanded pharynx of an infant [5]. However, current evidence and statistics confirm that some products larger than the size of the SPTF have caused choking.

One of the first studies evaluating the current size of the SPTF and the injuries and deaths of children due to the exposure of small parts was released more than two decades ago. Meyers and Bond (1989) evaluated a compilation of 195 choking incidents reported by the CPSC between July 1973 and May 1983, roughly a ten-year span, and found that 57% of the children's products whose size was available had diameters larger than the 1.25-inch standard [6]. Even with the statistics provided by Meyers and Bond, no changes have been proposed to the testing method since that time. It has been documented in subsequent studies that objects larger than the current size of the SPTF have caused choking following this article as manifested by the numerous recalls and injuries listed on the CPSC website, individual parent reports/complaints, and other related children's safety research projects [7, 8]. Other case examples of the hidden dangers posed by children's products have been reviewed in* Children and Injuries* by Frost, Ed.D. [9].

The objective of the present study is to provide the most up-to-date information on choking statistics and examples of children's products that are larger than the current fixture size that yielded a choking or related hazard. By providing these relevant statistics, it epitomizes that a larger test fixture could be the crucial next step in the prevention of choking occurrences.

In 2006, Playskool, Inc., voluntarily recalled their Team Talkin' Tool Bench after receiving reports of two boys under two years old who suffocated on the toy set's oversized nails, measuring 0.75 inches longer than the current SPTF size [10]. In 2007, an eight-year-old boy died after choking on a toy dart that measured 2.5 inches long and 0.75 inches wide, again, longer than the current SPTF size [11]. An 11-month-old boy also choked on a toy nail, part of the Little Tikes' Toy Tool Set, after it became forcefully lodged in his throat and prompted the original recall in 2009 [12]. Two additional choking incidents were reported even after the original recall of this product [13]. These are just a few examples of choking occurrences that resulted in injury and even death from children's products that measured larger in size than the current SPTF.

The current small part size may also not be adequately preventing children from choking due to the fact that, over the last five decades, children have grown taller and bigger in size. For example, according to Professor Mitch Blair, the average child's height has increased by 1 cm to 3 cm throughout every decade over the last 50 years. Thus, children on average have grown between 5 cm and 15 cm taller over the last five decades [14]. This means that one can assume that the child's organs, such as their esophagus, have grown exponentially with the height as well. Since the recommendation of the Academy of Pediatrics was made in 1987 regarding the size of an infant/toddler's esophagus to create the current SPTF size, there has been evidence that children have been growing taller since that time (roughly 30 years since the article was published) (see [6]). The current small part size has not adapted to the increase in size of children; thus a new and improved size (a larger one) may be suitable in preventing further choking incidents.

The present study, assessed by Kids In Danger (KID), sought to gather up-to-date information about the sizes of the products involved in the choking-related recalls in the past twelve years and whether the small parts test fixture is adequate in preventing choking and other related hazards.

## 2. Method

KID reviewed 303 recalled products publically listed by the CPSC that presented choking hazards to infants and young children due to small parts between January 1, 2003, and December 31, 2014 (This study is a thorough review of official recalls* reported* by the CPSC between January 1st, 2003, to December 31st, 2014. Individual incidents and complaints by parents are not included in this study.). Manufacturers tested these products and concluded that they were large enough and durable enough (i.e., would not break) for safe play prior to placement on the market. The products were eventually recalled due to breakage or even the original, intact product causing choking and related incidents. KID obtained the total number of recalled products and examined them by product categories and the kind of choking hazard caused by each product. Most importantly, KID focused on the sizes of the product or piece of the product that posed hazards and/or caused choking in proportion to the current size of the SPTF. Lastly, KID also reviewed the total number of incidents caused by the recalled products. Choking “incidents” include any interaction with the mouth, including mouthing, gagging, choking, ingestion, aspiration, or coughing of the product by the child. KID noted the various types of incidents that were caused by the products involved within the dataset, which are listed below. The authors of this article organized the data stated above in a spreadsheet (i.e., type of product and what kind of choking hazard the specific product caused), thus allowing ease of access to product details.

## 3. Results

Analyses show that 48.8% of the products within the dataset fall under the toys category (This study did not include outdoor toys such as bikes, battery-powered life-size electric vehicles, and trampolines.), 27.7% of the products fall under the clothing category (This study did not include glasses frames or sunglasses.), 18.9% of the products fall under the nursery category (This study did not include strollers, baby bouncers, exersaucers, furniture (cribs, sofas, and bean bag chairs), blankets, or high chairs.), and the final 4.6% of the products fall under the books and art supplies category. This categorizes the kinds of products that were listed on the CPSC website as having the potential to cause choking or have caused choking and related incidents.

With the current size of the SPTF, a notable quantity of the products within the dataset did not fit inside the cavity of the tube. Research shows that 17.1% of the products in the dataset were larger than the current fixture size and* still* posed a choking threat or caused choking and related occurrences in some instances. The additional 82.8% of the products in the dataset were smaller than the SPTF.

As described in Section 2, the next matter that KID analyzed was the number of incidents that occurred due to the reported products causing choking and related incidents. Analyses show that a total of 211 incidents were caused by these recalled products that occurred between January 1st, 2003, through December 31st, 2014. These incidents were caused by 63 of the 303 products, or 21% of the total products. Fifty-six of these 63 products were marketed for children under the age of three.  Table 1 categorizes the incidents by type.

Four of the choking incidents above resulted in death;* three* of the four deaths were caused by an object that was larger than the dimensions of the SPTF. Two of the deaths resulted in two children who choked on toy nails, as part of Playskool, Inc.'s Team Talkin' Toy Tool Bench, which measured 3 inches long. These toy nails were 0.75 inches longer than the current SPTF length.

## 4. Discussion

Statistics from the present study indicate that despite meeting the testing requirements via third-party testing prior to distribution, certain children's products still posed choking hazards and caused choking and even death in some instances. More research on the SPTF is necessary in order to determine whether the fixture should be enlarged. For instance, data from health care providers in the field and even complaints from parents/caregivers might indicate an even larger proportion of choking incidents than reported by the CPSC alone. With the data shown above, however, the current size of the SPTF may be one cause of not adequately preventing children from choking on children's products. Although there may be other factors involved, the SPTF was created with the sole purpose of preventing choking in children. Some products within the dataset that were larger than the SPTF have posed choking hazards and even caused choking and related injuries including death between 2003 and 2014, showing that its current size* may* not effectively prevent choking.

Overall, the current SPTF size has thus far not completely ruled out all choking and related hazards within children's products as exemplified through the numerous hazards, injuries, and deaths presented in this study. A larger test fixture would be the first step in generating the capacity to reduce the number of choking incidents in infants and toddlers. This is because it would abolish manufacturers from including parts even larger than the current size of a “small part” within their products. By doing so, it takes into account that small children can still potentially choke on small parts, but, by increasing the size of the SPTF, it can reduce the risk of choking and related hazards. A number of products within the dataset contained parts that were larger than the SPTF and still posed a choking threat. Therefore, a larger SPTF could theoretically rule out more potential risks and possibilities of choking.

## Supplementary Material

The original dataset includes 303 recalled products listed on the CPSC website which lists the date of original recall, the manufacturer, the number of reported injuries, and other pertinent information about each recalled children's product.

## Figures and Tables

**Table 1:** Type and occurrence of choking incidents.

	Percent (%)
Choking	46.91
Mouthing	27.96
Gagging	21.33
Ingestion	2.37
Coughing	0.94
Aspiration	0.50

## Additional Points

*Statement of Knowledge*. The SPTF has been implemented by the CPSC to measure for small parts and identify their presence on product labeling for the prevention of choking. Meyers and Bond (1989) discussed the inability of the SPTF to accurately prevent choking because objects larger than the SPTF have caused choking in children. No changes have been made by the CPSC to the small parts test fixture since the 1989 publication.* Statement of Addition to the Public*. This study provides up to date statistics showing that the current SPTF size does not adequately prevent choking. Three of the four deaths in this study were caused by an object that was larger than the current SPTF size. This study shows that enlarging the SPTF can help prevent choking in the future by eliminating slightly larger parts from children's products.
